# Supplementary material for: Cyathus striatus Extract Induces Apoptosis in Human Pancreatic Cancer Cells and Inhibits Xenograft Tumor Growth In Vivo
Source: Cancers (Basel). 2021 Apr 22;13(9):2017. doi: 10.3390/cancers13092017 (PMC8122434; doi:10.3390/cancers13092017)

HPAF-II cells with Actin (43kD)

PL45 cells with Actin (43kD)

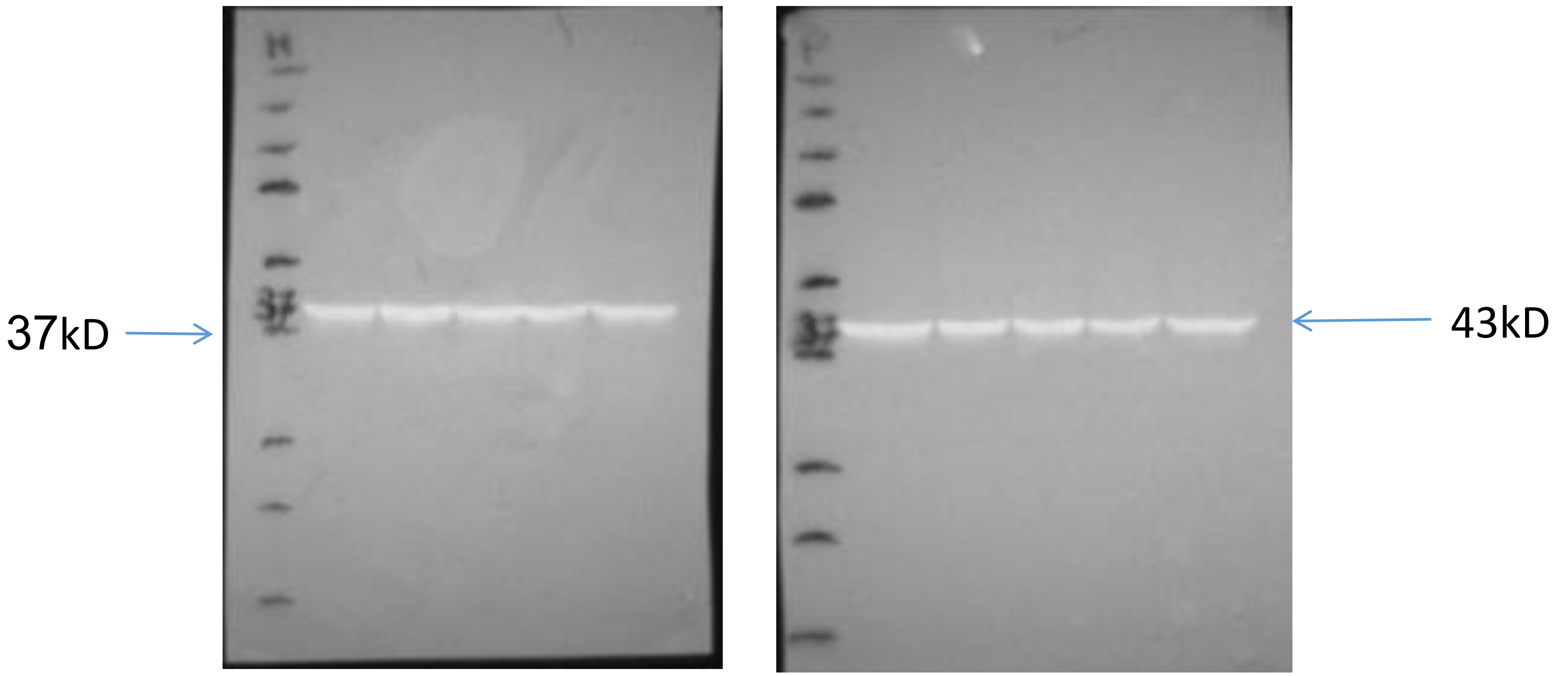

HPAF-II and PL45 cells with casp-3 Ab (32, 19, 17kD)

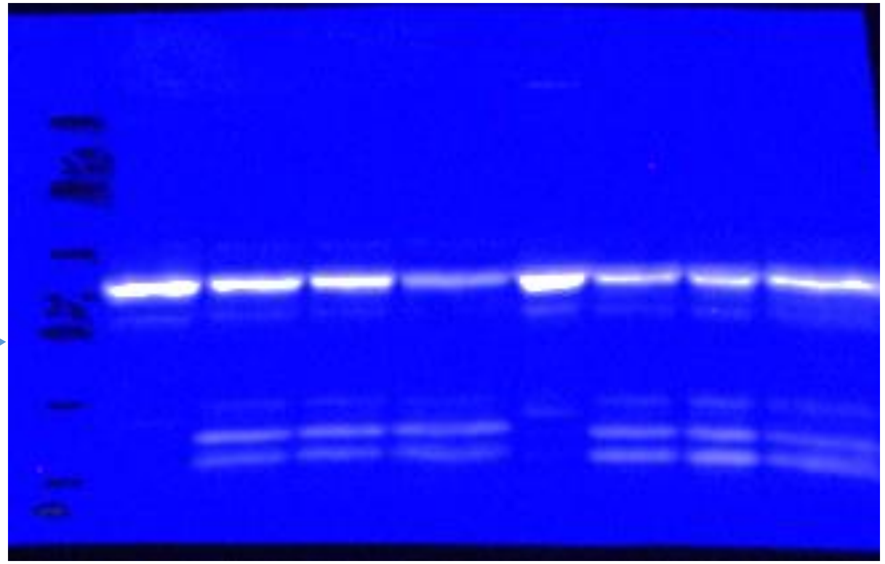

HPAF-II cells with casp-8 Ab (55, 43,41 kD)

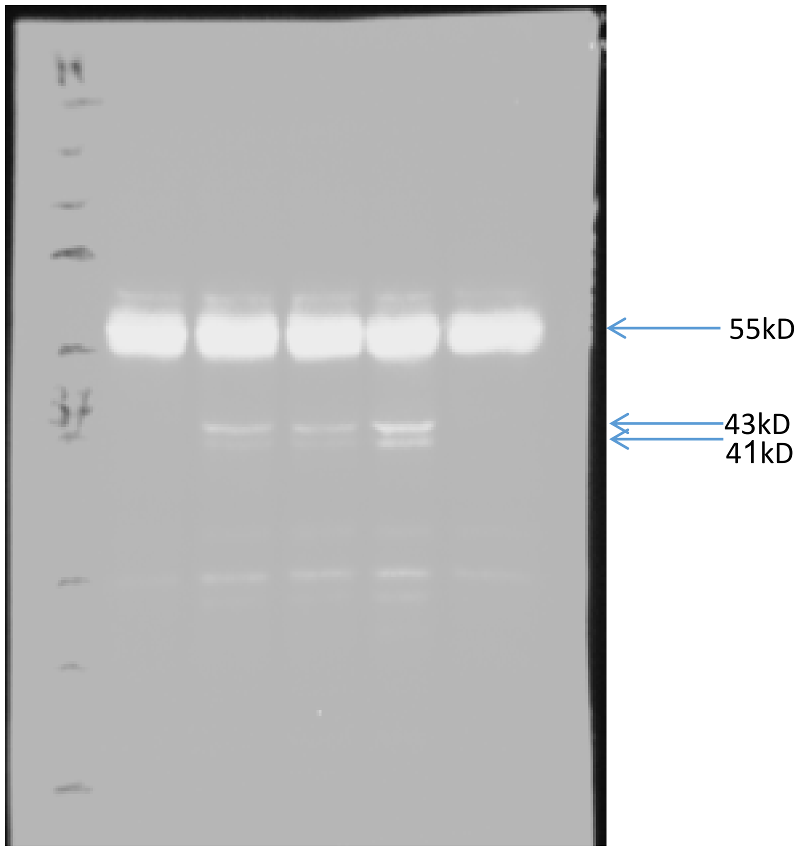

PL45 cells with casp-8 Ab (55, 43,41 kD)

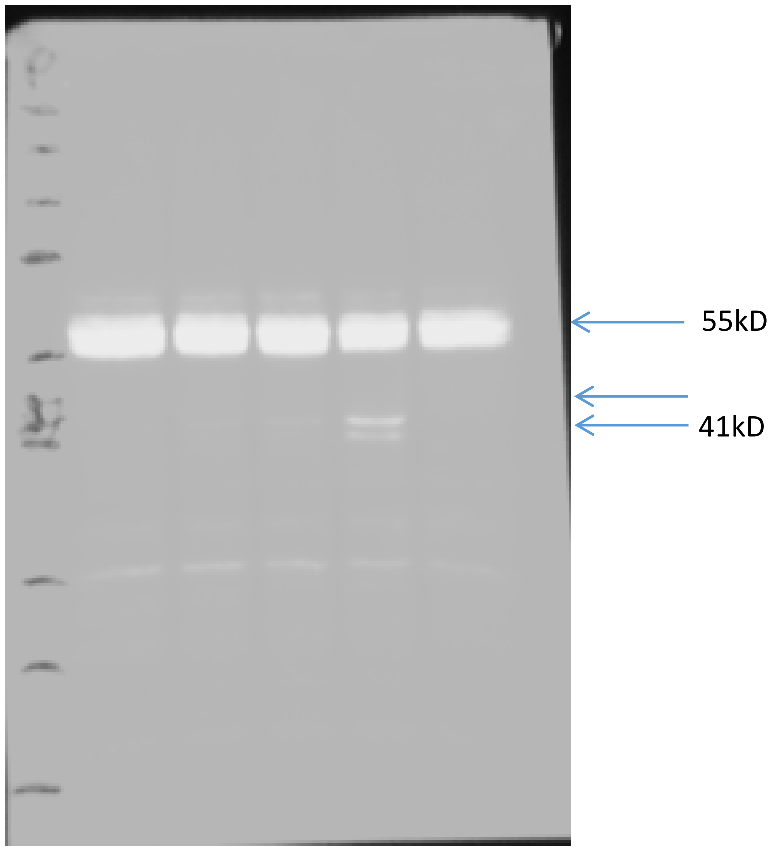

HPAF-II and PL45 cells with casp-9 Ab (47, 37, 35kD)

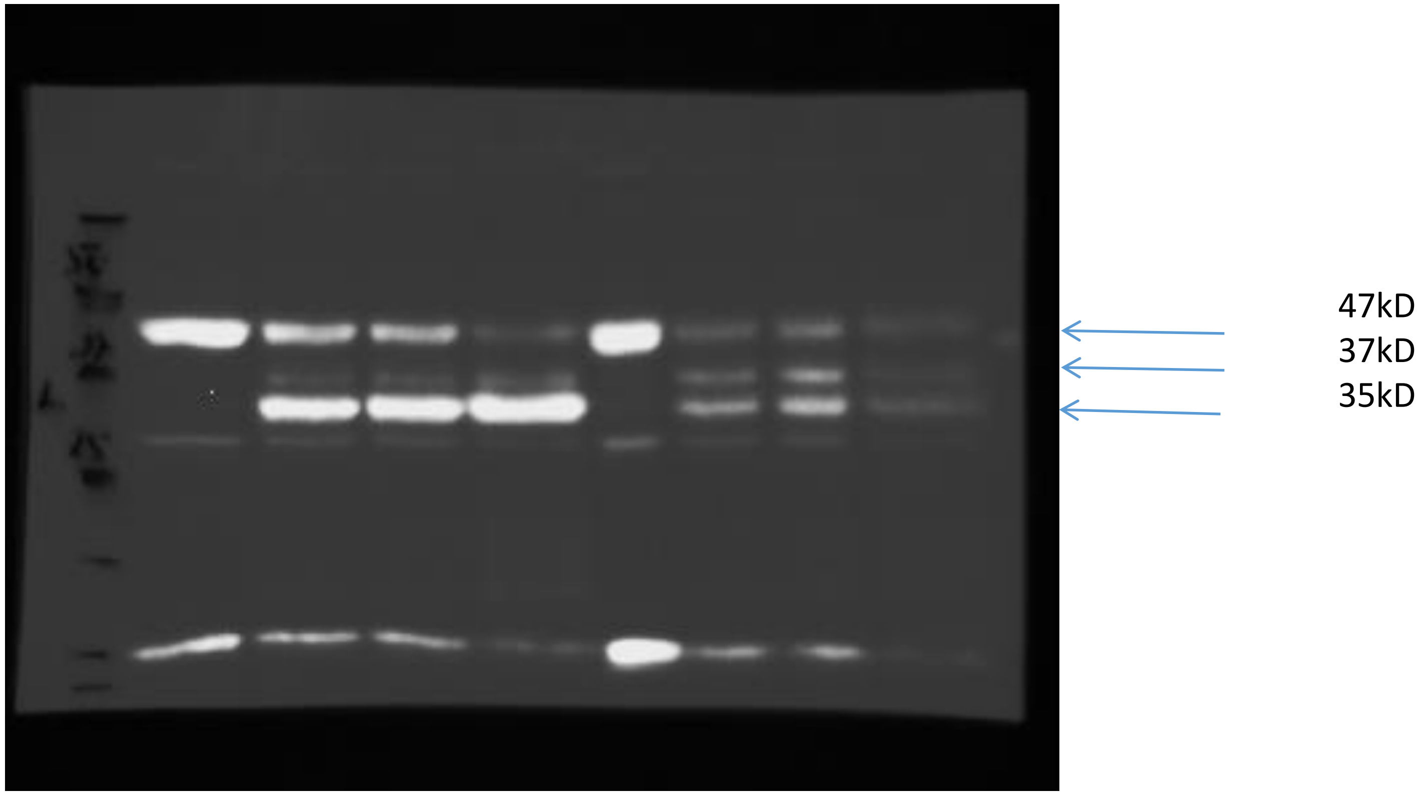

HPAF-II cells with PARP Ab (116, 89 kD)

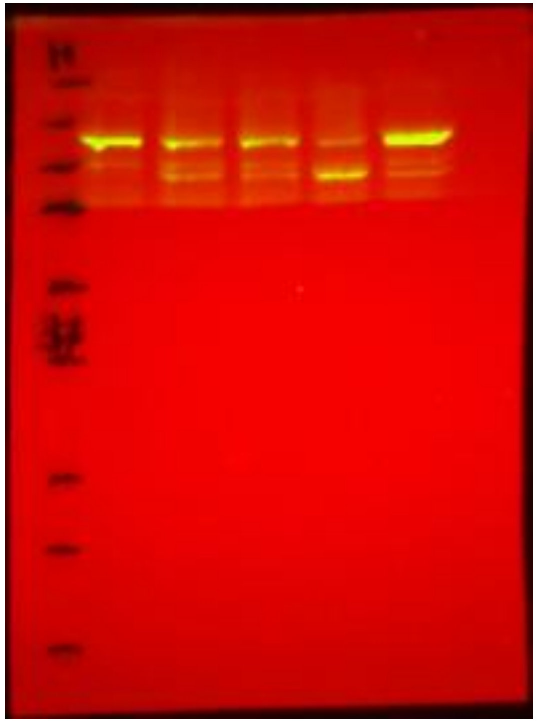

PL45 cells with PARP Ab (116, 89 kD)

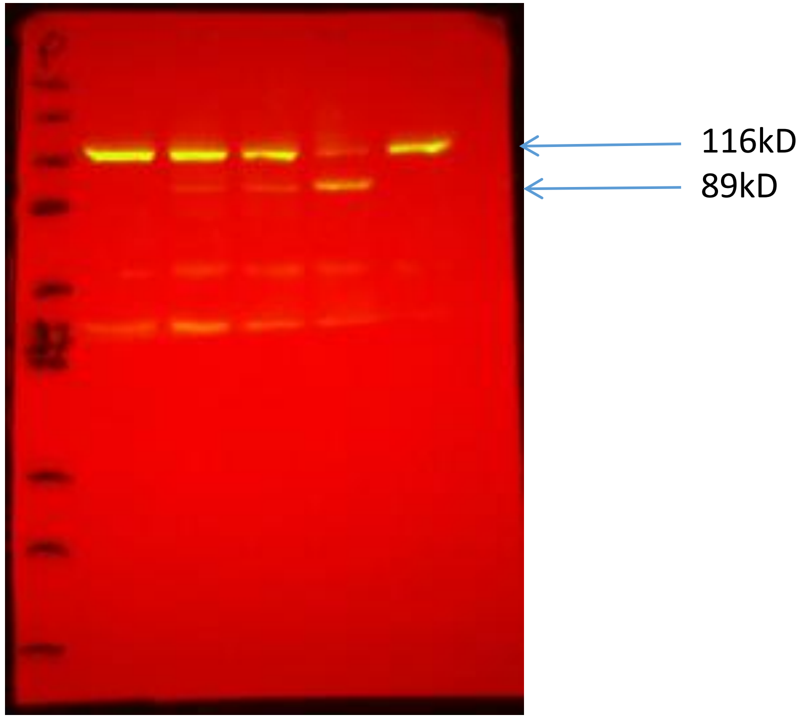

Supplement: Supplementary file 1 [file cancers-13-02017-s001.zip › cancers-1149791-original-images.pdf]
