# Supplementary material for: Cyathus striatus Extract Induces Apoptosis in Human Pancreatic Cancer Cells and Inhibits Xenograft Tumor Growth In Vivo
Source: Cancers (Basel). 2021 Apr 22;13(9):2017. doi: 10.3390/cancers13092017 (PMC8122434; doi:10.3390/cancers13092017)
Supplement: Supplementary file 1 [file cancers-13-02017-s001.zip › cancers-1149791-supplementary.pdf]

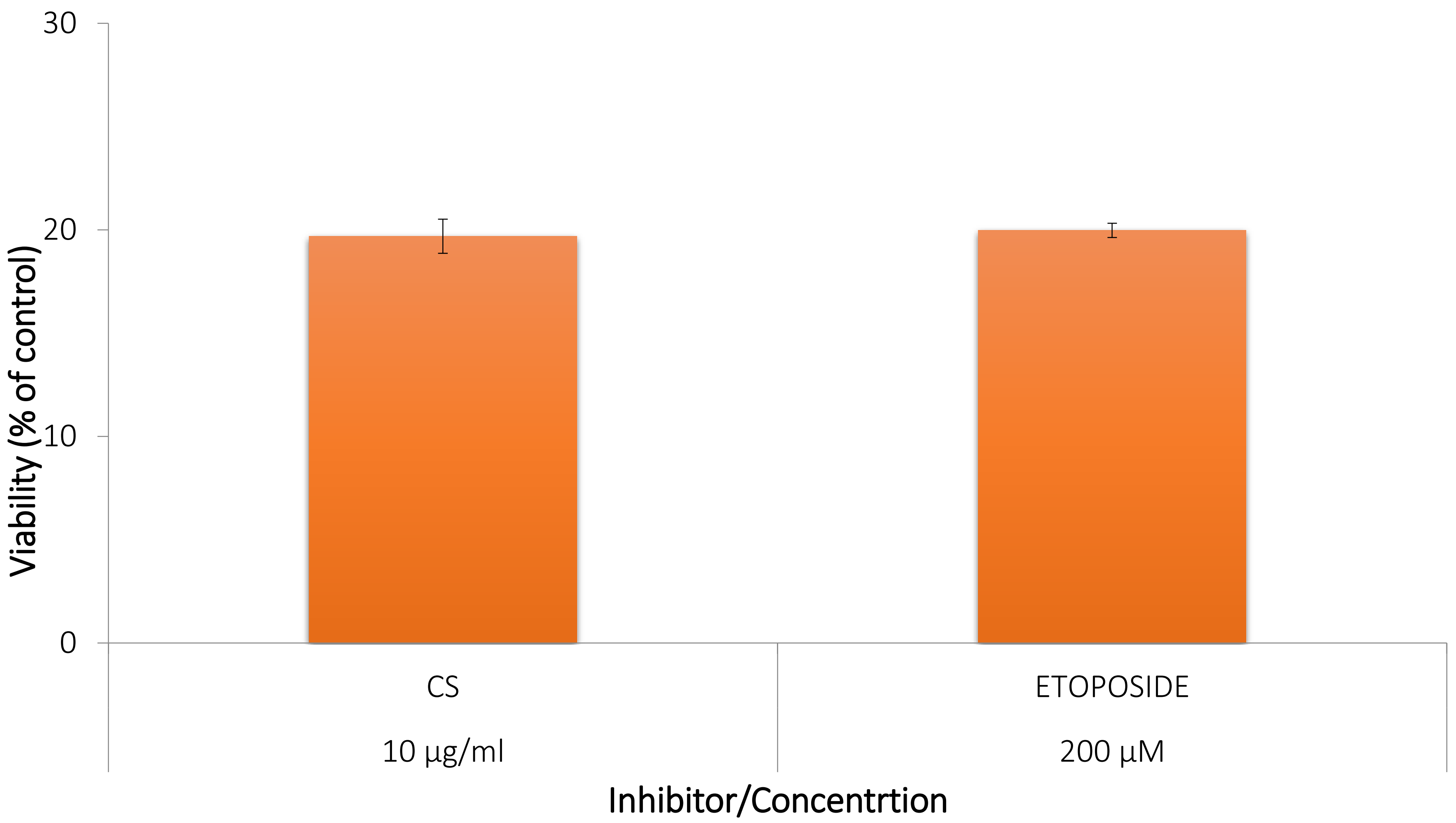

The effect of CS on cell viability of NHDF (normal human dermal fibroblasts) cells.

NHDF normal human fibroblast cells were treated with 10 µg/ml or 200 µM etoposide for 24 h followed by cell viability assessment using XTT assay as described under "Materials and Methods". Etoposide, is a chemotherapy medication used for the treatments of a number of types of cancers.
